# Chemically Enhanced Peptide and Protein Therapeutics

**DOI:** 10.3390/pharmaceutics15030827

**Published:** 2023-03-03

**Authors:** Cristina Díaz-Perlas, Benjamí Oller-Salvia

**Affiliations:** Institut Químic de Sarrià (IQS), Universitat Ramon Llull, 08017 Barcelona, Spain

Proteins and peptides are on the rise as therapeutic agents and represent a higher percentage of approved drugs each year: 24% in 2021 vs. 13% in 2016 [1]. These classes of therapeutics can engage targets considered undruggable by small molecules. However, proteins and peptides are often less stable and labile to proteolysis, which limits their effective use as therapeutics. These challenges can be overcome by chemical modification or “chemical enhancement”. For instance, peptide lability to proteases can be greatly reduced by cyclization or the use of non-canonical amino acids, while clearance may be decreased by conjugation to albumin. Proteins may also be chemically enhanced; for instance, stability may be increased by conjugating polyethylene glycol and the targeting capacity of antibodies may be combined with the potency of toxic small molecules by chemical conjugation. In this Special Issue, we include some of the latest advances in this thriving field.

Peptides have many therapeutic applications, including as antimicrobial agents and for the treatment of diabetes, gastrointestinal disorders, and tumors, among others. Moreover, peptides can also be used to endow targeting or cell penetration properties to other molecules. The use of peptides as therapeutics is growing, and a total of 33 non-insulin peptide drugs have been approved worldwide since 2000 [2]. More than 170 peptides are in active clinical trials, with many more in preclinical development. Peptides have two main physiological disadvantages: rapid clearance and high lability to proteases. The first limitation may be overcome by conjugation to proteins or polymers, while the latter may be addressed by cyclization, backbone modification, and the use of non-canonical side-chains. Chemical modification can not only be used to increase peptide residence time and resistance to proteases, but also to boost binding and activity. Since peptides have less than 40 amino acid residues (as defined by the FDA) and are amenable to chemical synthesis, introducing chemical modifications to enhance their properties is readily accessible.

This Special Issue contains several publications showing a wide variety of strategies to enhance peptide activity. As Shatz-Binder and collaborators describe [3], in order to decrease blood clearance, therapeutic peptides may be conjugated to polymers or proteins such as albumin, antibody crystallizable fragments (Fc), or nanoparticles. Increasing peptide residence time and their accessibility for immune recognition is key in peptide vaccines. For instance, Skwarczynski, Toth, and collaborators prepare an anticancer vaccine by conjugating a peptide derived from the human papilloma virus, responsible for cervical cancer, to a polyleucine tail [4]. They incorporate the chemically enhanced peptide into liposomes and show that their vaccine induces a strong cellular immune response in tumor-bearing mice.

Focusing more on protease lability, Andreu’s group provide us with a comprehensive review on chemically enhanced peptides as modulators of G-protein-coupled receptors (GPCR) with drug-like properties. They focus on GPCR-disrupting peptides and derivatives with improved potency and bioavailability [5]. Cyclization of peptides has extensively been used to confer higher protease stability and affinity due to reduced binding entropy [6,7]. In this Special Issue, Rizwanul Haq and collaborators investigate the mechanism of resistance of colistin, a cyclic antimicrobial peptide with a C-terminus-to-side-chain connectivity [8]. They find that electrostatic forces are key in preventing interaction with the cell membrane, and this hinders the internalization of the antimicrobial peptide (AMP) into bacteria. Rabanal and co-workers focus on improving another antimicrobial peptide with a C-terminus-to-side-chain cyclization, polymyxin B. The antibacterial activity is fine-tuned by utilizing non-canonical side-chains to redistribute the hydrophobicity within the scaffold [9]. The authors show that by introducing small changes, such as the replacement of leucine by norleucine, safer and more selective antibiotics can be obtained. Gomes et al. also study the possibility of adding imidazole moieties to enhance the activity of an antibacterial peptide and disclose a promising lead to tackle complicated skin infections with a new chimeric peptide that combines both collagenesis-boosting and antimicrobial properties (PP4-3.1) [10]. Aiming to generate even more accessible therapeutics, Mor’s laboratory studies a peptidomimetic antibiotic with a completely altered backbone, combining positively-charged amino acids and aliphatic methylene chains [11]. The authors of this study find that this peptidomimetic acts through a similar mechanism to other AMPs but with a significantly higher potentiation capacity. Interestingly, amino acids alone may also boost the activity of other materials. For instance, El-Fakharany and collaborators show that decorating tungsten oxide nanoparticles with Cys or Trp boosts their broad-spectrum antibacterial activity [12].

Other important roles of peptides in biotherapeutics is their capacity to provide selectivity by targeting specific membrane proteins, to enhance cell internalization or to increase transport across biological barriers. In this Special Issue, Villaverde and coworkers demonstrate efficient antitumor activity of a construct containing a peptide targeting anti-CXCR4 fused to GFP that is covalently conjugated to a microtubule polymerization inhibitor, monomethyl auristatin E [13]. When targeting proteins or nanoparticles, clearance is generally not an obstacle because the peptide is conjugated to a large cargo, reducing glomerular filtration. However, lability to proteases may decrease targeting or transport capacity. In this Special Issue, we have reviewed the latest advances in the field of protease-resistant targeting and cell-penetrating peptides, critically assessing the advantage of enhancing the metabolic stability of these peptides [14]. Two main approaches are used to make these peptides less prone to protease degradation: enantio/retro-enantio isomerization [15,16] and cyclization [17,18].

In proteins, the most extended modification is PEGylation, which reduces protein aggregation, immune recognition, protease degradation, and renal clearance [3]. Chemical enhancement has also been used to boost protein function, such as increasing enzyme activity with non-canonical amino acids [19], improving thermal stability in proteomimetics [20], and generating targeted conjugates. In this Special Issue, Kogan and collaborators describe the use of the ferritin protein as a coating to efficiently deliver gold nanoparticles into cells [21]. These nanoparticles have great potential as theranostic agents but cannot be injected without modification due to toxicity and specificity issues. In this article, they use ferritin homopolymers as nanoreactors to synthesize gold nanoparticles, and they find low cytotoxicity and improved cellular uptake in cells with high levels of the transferrin receptor.

The most extensively utilized targeted therapeutic conjugates are antibody–drug conjugates (ADCs). ADCs couple the high selectivity of the antibody to the high potency of the antineoplastic toxin. An update on the current trends of this class of chemically enhanced antibodies is also presented in the review by Shatz-Binder and collaborators [3]. A key parameter in the development of ADCs is the linker between the antibody and the cytotoxic agent, as its characteristics impact the efficacy and pharmacokinetics of the drug. The numerous types of linkers, including cleavable to non-cleavable ones, are summarized and compared in a review by Albericio’s group [22]. Site-specific modification has also been shown to increase the therapeutic index of ADCs [23]. This can be achieved by a variety of chemogenetic approaches, including enzymatic ligations, genetic encoding of cysteines, and non-canonical amino acid in the sequence [24,25,26]. Other opportunities for chemically enhancing antibodies lie in the raising field of conditionally active antibodies [27].

In conclusion, this Special Issue covers many of the latest advances in chemically enhanced peptides and proteins, from conjugation improving selectivity or decreasing renal clearance to cyclization and the use of backbone and site–chain modifications to increase protease resistance and affinity. A number of applications are covered, from GPCR modulation to cancer treatment, with a special focus on antimicrobial peptides. Overall, this Special Issue will provide the reader with a collection of recent scientific advances in the development of safer and more efficacious peptide and protein therapeutics utilizing chemical tools.
